# A Hybrid Intelligent Simulation System for Building IoT Networks: Performance Comparison of Different Router Replacement Methods for WMNs Considering Stadium Distribution of IoT Devices

**DOI:** 10.3390/s22207727

**Published:** 2022-10-12

**Authors:** Admir Barolli, Shinji Sakamoto, Kevin Bylykbashi, Leonard Barolli

**Affiliations:** 1 Department of Information Technology, Aleksander Moisiu University of Durres, L.1, Rruga e Currilave, 2001 Durres, Albania; 2 Department of Information and Computer Science, Kanazawa Institute of Technology (KIT), 7-1 Ohgigaoka, Nonoichi 921-8501, Japan; 3 Department of Information and Communication Engineering, Fukuoka Institute of Technology (FIT), 3-30-1 Wajiro-Higashi, Higashi-Ku, Fukuoka 811-0295, Japan

**Keywords:** Internet of Things, wireless mesh networks, particle swarm optimization, simulated annealing, distributed genetic algorithm

## Abstract

As the Internet of Things (IoT) devices and applications proliferate, it becomes increasingly important to design robust networks that can continue to meet user demands at a high level. Wireless local area networks (WLANs) can be a good choice as IoT infrastructure when high throughput is required. On the other hand, wireless mesh networks (WMNs), which are WLANs with mesh topology following the IEEE802.11s standard, have many advantages compared to conventional WLANs. Nevertheless, there are some problems that need solutions. One of them is the node placement problem. In this work, we propose and implement a hybrid intelligent system that solves this problem by determining the position of mesh nodes by maximizing the mesh connectivity and the coverage of IoT devices. The system is based on particle swarm optimization (PSO), simulated annealing (SA), and distributed genetic algorithm (DGA). We compare the performance of three router replacement methods: constriction method (CM), random inertia weight method (RIWM), and rational decrement of Vmax method (RDVM). The simulation results show that RIWM achieves better performance compared to CM and RDVM because it achieves the highest connectivity while covering more clients than the other two methods.

## 1. Introduction

With the rapid development of communication technologies, the number of the Internet of Things (IoT) devices is increasing exponentially, which, in turn, has increased the demand for wireless connectivity. There are many ways to provide wireless network connections for IoT devices, including wireless local area networks (WLANs), Bluetooth, ZigBee, radio frequency identification (RFID), and low-power wide-area networks (LPWA). LPWA is one of the most efficient technologies because it can reduce the power consumption of IoT devices while covering a wider area compared to the other wireless technologies [1].

However, the throughput of LPWA is lower than the other technologies, so WLANs can be a good choice when high throughput is required. Nowadays, WLANs have enough specifications to adapt not only for small, high-speed networks but also for IoT networks. For example, IEEE802.11ax can save energy consumption using target wake time technology [2]. Additionally, the communication distance of WLANs in IEEE802.11ax becomes longer compared with conventional standards [3,4]. Moreover, the communication distance of WLANs is now also suitable for IoT. For example, IEEE802.11ah, which is a long-range WLAN, has extended communication distance to 1 km [5,6]. In particular, the wireless mesh networks (WMNs), which are WLANs with mesh topology specified previously by the IEEE802.11s standard and currently included in IEEE802.11, can be a good choice for IoT because they offer high scalability, high robustness, high maintainability, and high-speed connection. WMNs can be used as last mile networks and for wireless sensor network (WSN) applications in medical, transport and surveillance systems in urban areas, metropolitan areas, neighbor communities and municipal area networks, among others.

Considering WSN applications, the WMN nodes can serve as sink nodes collecting sensed data from sensors monitoring a wide area and even as sensing devices. Each sink node is connected to other sink nodes via wireless links, enabling the transmission of the information to the internet or to other networks over multiple paths. As a result, the failure of a sink node does not disrupt the data collection and transmission, making the network more robust and reliable [7]. The collected data can be analyzed and processed by a wireless sensor actor network (WSAN), which can have the actors perform various tasks based on the application and the outcome of data analysis.

**Figure 1:**
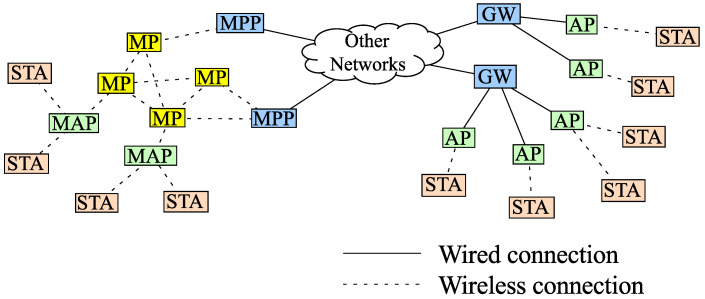
WMNs (**left**) vs. conventional WLANs (**right**).

The nodes of a WMN can be categorized into four types based on the role they play in the network [8]: mesh points (MP), mesh access points (MAP), mesh portal collocated with MP (MPP), and stations (STA). The STA is the client node of conventional WLANs. The MP supports the peer link management protocol (PLMP), but it does not enable Internet connection to the STA [9] because the Internet connection is provided by the MAP. The MAP in WMNs can be considered as an MP with the capabilities of providing Internet connection to the STA. The MPP is a node with a gateway feature, which is connected to other networks via cables. Compared with WMNs, a WLAN needs more cables, as shown in Figure 1; hence management cost and up-front cost are much higher than the costs required to deploy a WMN.

Despite their flexible architecture and numerous advantages, WMNs face problems of their kind. One of the major problems is related to the placement of the nodes in the network because their placement affects the connectivity and user coverage. The designers of the WMNs must place the nodes in positions that enable high connectivity and full coverage, but solving this bi-objective optimization problem is not straightforward at all as it belongs to a class of problems known as NP-hard problems.

In our previous work, we proposed two simulation systems for solving the node placement problem in WMNs: a particle swarm optimization (PSO) and simulated annealing (SA)-based simulation system called WMN-PSOSA [10] and a genetic algorithm (GA)-based system called WMN-GA [11].

In this paper, we propose and evaluate a hybrid intelligent system based on PSO, SA and distributed GA (DGA) called WMN-PSOSA-DGA.

We consider different router replacement methods for each distribution of IoT devices in order to find good solutions for different scenarios. In our previous work [12], we considered normal, Weibull and Boulevard distributions of mesh clients and presented the results for five router replacement methods. In this paper, we consider the Stadium distribution and present a comparison study not only for different router replacement methods but also for roulette wheel and random selection methods.

The contributions of this work are as follows.

- We implement a hybrid intelligent system that is based on three intelligent algorithms: PSO, SA, and DGA.
- We present a comparison study for three different router replacement methods.
- We consider the Stadium distribution of IoT devices in order to find good solutions for similar real-life environment scenarios.

The remainder of the paper is as follows. In Section 2, we present a literature review. We explain our hybrid simulation system in Section 3. The simulation results and discussions are given in Section 4. Finally, we provide conclusions and discuss future work in Section 5.

## 2. Related Work

There are many research works for node selection and routing in WMNs. L. Zhao et al. proposed a relay node selection model for WMNs in community use called community wireless mesh networks (CWMNs) [13]. The model is focused on supporting QoS and can be built on top of existing standards. Y. Rao et al. proposed a QoS-based mobility management (QBMM) algorithm using a metric calculated by expected transmission count [14]. By considering mobility, the relay node selection problem becomes more difficult. They evaluated the proposed QBMM algorithm by comparing it with the pointer forwarding algorithm (PFA) and without PFA (WPFA). The authors concluded that the QBMM has the best performance compared to the PFA and WPFA algorithms. Z. Kuang et al. proposed two routing algorithms [15]: a high-reliability, low-latency wireless link weight computing algorithm (RL2W) and a high-reliability, low-latency routing and spectrum allocation algorithm based on dynamic programming in CWMNs (HRL2A). Simulation results show that HRL2A performs better than conventional algorithms in the considered scenario.

Another problem of WMNs is the resource allocation problem. For instance, the channel assignment in WMNs is a crucial issue because it directly affects the performance of WMNs. Thus, MAC protocols for WMNs considering channel assignment are developed [16,17]. In [18], the authors develop allocation scheduling. J. Gui et al. classified the channel assignment problem into centralized and distributed problems [19]. For solving the channel assignment problem in WMNs, A. P. Subramanian et al. designed a distributed algorithm [20]. They obtained lower bounds on the overall network interference by considering the channel assignment problem as a semi-definite program and a linear programming formulation of their optimization problem. Y. Zhao et al. focused on resource allocation in WMNs considering multi-channel and multi-radio approaches [21]. They proposed an optimization model which formulates each phase as a mixed integer linear problem. The model is integrated with local search to reduce the computational complexity of the optimization.

To solve NP-hard problems, the heuristic approaches are widely used, as described in [22]. Moreover, V. Kesavan et al. described that most researchers mainly focus on single optimization problems because multi-optimization results in poor performance [23].

Amaldi et al. [24] proposed optimization models based on mixed-integer linear programming (MILP) that minimize the network installation cost while providing full coverage to wireless mesh clients. The proposed models allow the selection of the number and the positions of mesh routers while considering the state of different network parameters, such as traffic flow, channel assignment, rate adaptation, and interference. Because the mixed linear programming models need time to provide a good solution, the authors proposed relaxation-based heuristic algorithms that jointly solve this problem.

Since time is nearly always a restricting factor, many authors have proposed approaches based only on heuristic methods. In [25], the authors proposed a new GA approach that maximizes network connectivity and coverage area. The authors stated that the proposed approach can efficiently provide optimal solutions for the mesh router placement problem. The simulated annealing (SA) approach was employed in [26] to find the locations of routers so that all clients located in an area of interest are covered. The SA is compared with a new approach that the authors propose, called SA-based center of mass (SAC), and the results show that SAC outperforms the classic SA algorithms. The authors state that SAC can improve the network and the quality of solutions in just about half the time needed for a classic SA to converge.

Other authors have optimized the coverage and connectivity by means of the PSO algorithms. In [27], the authors proposed an approach that can solve the problem of overlapping and coverlessness. The proposed approach can escape the local minima, thus improving the quality of solutions and convergence speed. Nouri et al. [28] showed that not only is the accelerated PSO algorithm faster and less complex, but it also obtains better solutions than linearly decreasing weight PSO (LDWPSO). The authors in [29] take into account the social relationship among the clients and present an algorithm that can dynamically adapt to the social community behavior. The results show that the social-based PSO algorithm increases the coverage of mesh clients and improves the network connectivity in dynamic social scenarios.

Table 1 summarizes the aforementioned research papers whose research work is in the same line of work as this paper. However, we consider a hybrid intelligent algorithm composed of three intelligent algorithms, which is more challenging but overcomes the drawbacks of single algorithm approaches.

## 3. Proposed and Implemented Simulation System

This section describes the implemented hybrid intelligent system based on PSO, SA and DGA to solve the node placement problem in WMNs.

We define a rectangle area to deploy mesh nodes. The mesh nodes consist of mesh routers and mesh clients. In the considered area, M mesh router nodes and N mesh client nodes are located in arbitrary positions in the considered rectangle area.

The objective is to find the mesh routers’ positions that maximize the connectivity and user coverage. We try to maximize those two metrics simultaneously. The connectivity is measured by the size of the giant component (SGC) [30], which is the number of connected mesh routers. The user coverage is measured by the number of covered mesh clients (NCMC) by routers. The SGC and NCMC affect the WMN’s performance directly, so these metrics are very important.

In this work, we propose and implement a hybrid intelligent simulation system based on particle swarm optimization (PSO), simulated annealing (SA), and distributed genetic algorithm (DGA) in order to solve the node placement problem in WMNs.

### 3.1. PSO and SA

The PSO is a multi-agent-type meta-heuristic algorithm inspired by the behavior of insects and schools of fish [31]. Each particle follows a simple rule, yet PSO searches for solutions in the entire search space through all particles. Each particle shares the information with the other particles during the optimization. Then, all particles move to better points in the solution space by following a simple rule.

Each particle has a position in the solution space, a velocity, and a fitness value. The fitness value of each particle is calculated from its position and velocity. The velocity in each iteration is decided using the previous velocity, the best solution found by all particles, and the best solution found by the particle itself during the previous iterations.

The PSO generates the initial parameters randomly. After that, it keeps following a simple rule and updates its velocity, until the termination condition is satisfied. Finally, the PSO gives the best solution found.

SA is one of the local search-based optimization algorithms having a mechanism for escaping from local optima [32]. SA uses a temperature parameter for accepting a worse solution. Accepting worse solutions allows SA to avoid getting trapped in local optima, which makes SA algorithms capable of searching for solutions in the entire space.

SA accepts a worse solution via a probabilistic process. The probability of acceptance *P* is defined by using the SA temperature value *T* as:

(1) $$\begin{array}{cc} P & =\min\left(1,\exp\left[\frac{f\left(s^{\prime }\right)-f\left(s\right)}{T}\right]\right), \end{array}$$

where $s^{\prime }$ is the neighboring solution, *s* is the current solution, and *f* is the fitness function. In this case, we consider that the higher the fitness value, the higher the chances of finding a better solution.

The SA temperature value is set high at the beginning of the search, and it decreases as the search continues. This means the acceptance probability for a worse solution decreases with the number of iterations. This mechanism is known differently as the cooling schedule. In our system, the next temperature value is calculated as $T_{n+1}=\alpha \times T_{n}$. We set the SA starting temperature, the SA ending temperature, and the number of iterations. Therefore, we can calculate α as

(2) $$\alpha ={\left(\frac{\mathrm{SA}\;\mathrm{ending}\;\mathrm{temperature}}{\mathrm{SA}\;\mathrm{starting}\;\mathrm{temperature}}\right)}^{1.0/\mathrm{number}\;\mathrm{of}\;\mathrm{iterations}}.$$

It should be noted that when the solution *s* is not updated, the only parameter that changes is the velocity, whereas the positions retain their values.

**Figure 2:**
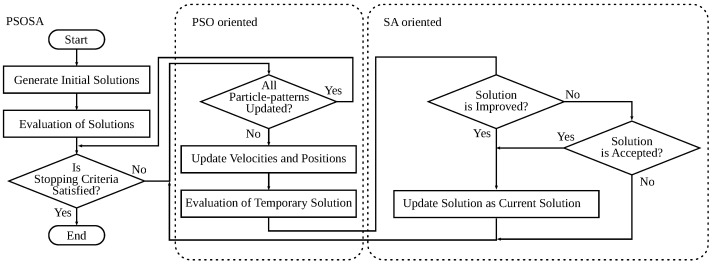
Flowchart of PSOSA.

In our system, SA is used in combination with PSO. One of the most critical decisions of local search-based optimization algorithms is whether to accept a neighboring solution or not since there is no guarantee that the solution leads to the best solution in the search space. Therefore, to increase the probability that a neighboring solution can lead to the best solution, we use as the SA’s neighboring solutions the solutions found by the PSO part. The flowchart of PSOSA is shown in Figure 2.

### 3.2. DGA

The GA is a method that imitates the evolution of species [33]. The basic idea is that the species suitable for the present environment tend to survive to the next generation. To apply this mechanism for optimization, the GA models the solutions as individuals represented as a gene. An individual (gene) has a fitness value, which should have a higher value so it can be considered a better solution.

To find a better solution, GA has some operations such as selection, crossover, and mutation. The selection operation chooses the genes for the next generations. There are some methods for selection, such as tournaments and roulette wheel. The crossover operation creates a new gene from two different genes. The mutation operation creates a new gene by changing the gene information. The GA runs operations probabilistically to create the next generation.

**Figure 3:**
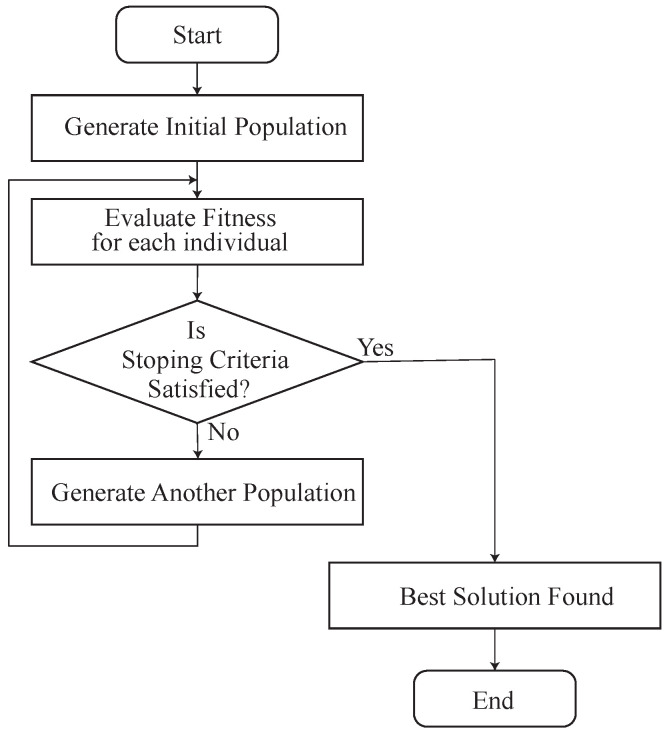
Flowchart of DGA.

DGA is an improvement of GA. DGA prepares some islands for independent evolution. When genes move to another island, the process is known as migration. Migration is an idea for obtaining diversity so that the search space is widely explored. The flowchart of DGA is shown in Figure 3.

### 3.3. WMN-PSOSA-DGA

#### 3.3.1. Fitness Function

The fitness function is defined by:

(3) Fitness=α×SGC+(1−α)×NCMC

where α is a weight coefficient decided by users. The fitness function defines the fitness value from the positions of mesh nodes and their communication distance. We evaluate a solution by comparing the fitness value for different solutions and check whether the solution is improved or not.

#### 3.3.2. PSOSA Implementation

The PSOSA part of WMN-PSOSA-DGA decides the next positions by using the velocity parameter. The velocity is updated every iteration. The velocity update method is called the router replacement method. There are several router replacement methods proposed in the literature. In this paper, we consider the following three methods.

Constriction Method (CM): CM is a method in which the PSO parameters are set as: ω=0.729, $C_{1}=C_{2}=1.4955$. This is based on analysis of PSO [34].

Random Inertia Weight Method (RIWM): In RIWM [35], the ω parameter changes randomly from 0.5 to 1.0. The $C_{1}$ and $C_{2}$ are kept at 2.0. The average of ω is estimated to 0.75.

Rational Decrement of Vmax Method (RDVM): In RDVM [36], PSO parameters are set to an unstable region (ω=0.9, $C_{1}=C_{2}=2.0$). The $V_{max}$ decreases with the number of iterations as shown in the following equation.

(4) $$V_{max}\left(x\right)=\sqrt{W^{2}+H^{2}}\times \frac{T-x}{x}$$

where *W* and *H* are the width and the height of the considered area, *T* is the total number of iterations, and *x* is the current number of iterations.

When a solution evaluated by the fitness function improves, PSOSA accepts the solution as the new current solution. In the case that the evaluated solution worsens, PSOSA uses the SA mechanism.

#### 3.3.3. DGA Implementation

The DGA part of WMN-PSOSA-DGA considers WMN as a gene. DGA tries to find better positions of mesh routers by applying operations such as selection, crossover, and mutation.

The DGA calculates the fitness value by considering the communication distance and the positions of mesh nodes. Then, DGA conducts the following operations probabilistically every iteration until the termination condition is satisfied.

The selection is an operation in which a gene with a higher fitness value is selected for the next generation. However, always surviving genes with higher fitness values often results in a premature convergence of the algorithm. To avoid this early convergence, many methods have been proposed in the GA field. In WMN-PSOSA-DGA, we implement the roulette wheel method. The roulette wheel method is a selection method that uses fitness value as a probability. A gene having a higher fitness value tends to be selected for the next generation with a higher probability, but the probability is never 1 even for the gene with the highest fitness value.

The crossover operation produces a new gene from two selected genes. There are many crossover methods [37,38,39], such as blend crossover (BLX-α), unimodal normal distribution crossover (UNDX), multi-parental UNDX (UNDX-m), and simplex crossover (SPX). In this work, we use the SPX crossover operation [40,41]. To solve an *n* dimensional problem, SPX uses n+1 parents. Firstly, SPX calculates the center of gravity $x^{p}$ from the parents as follows:

(5) $$\begin{array}{c} x^{p}=\frac{1}{n+1}\sum_{k=1}^{n+1}x^{k}. \end{array}$$

Then, $y^{i}$ is defined as:

(6) $$\begin{array}{c} y^{i}=x^{p}+\epsilon \left(x^{i}-x^{p}\right) \end{array}$$

where ϵ is the extension rate, which is a real number larger than 1 but generally set to $\sqrt{n+2}$. Here, a new gene $x_{c}^{i}$ can be created by the following formula:

(7) $$\begin{array}{c} x_{c}^{i}=\left\{\begin{array}{cccc} \; & y^{1} & \; & \left(i=1\right)\\ \; & y^{i}+\xi_{i}\left(x_{c}^{i-1}-y^{i}\right) & \; & \left(i=2,3,\cdots ,n\right) \end{array}\right. \end{array}$$

where $\xi_{i}$ is an independent random number for each *i*, which follows the normal distribution $N(0,\sigma_{\xi }^{2})$.

The mutation operation changes a piece of the gene’s information. If the mutation rate is high, the GA behaves as a random search. Therefore, a low mutation rate that is higher than zero is widely accepted as a best practice. The reason why a gene must mutate is that it allows the algorithm to escape from local optima. It should be noted, however, that mutation occurs for only one gene, and generally, the mutation operation does not directly affect the other genes. There are different mutation methods proposed for GA. In this paper, we consider the boundary mutation method, which generates only the minimum or the maximum value of a feasible range of values.

#### 3.3.4. Migration

**Figure 4:**
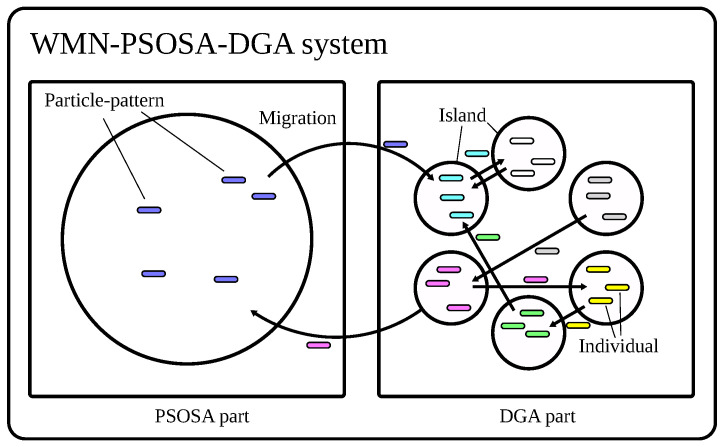
Model of WMN-PSOSA-DGA migration.

**Figure 5:**
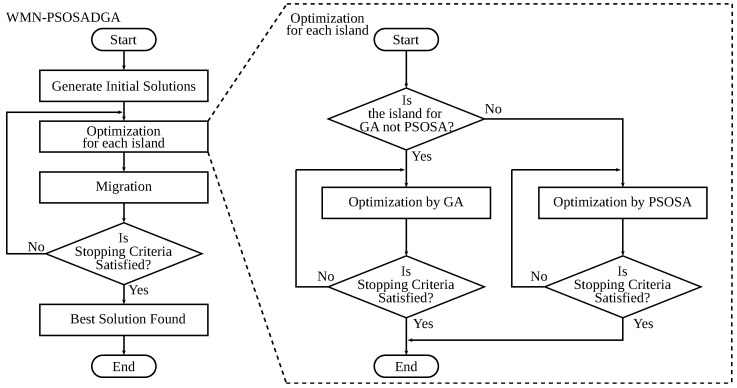
Flowchart of WMN-PSOSA-DGA.

The model of WMN-PSOSA-DGA migration is shown in Figure 4. The processes of the PSOSA part and the DGA part are independent from each other. The implemented WMN-PSOSA-DGA hybrid intelligent system considers islands to enhance the diversity of solutions. The migration operation migrates a solution (particle pattern or individual) to other islands. The operations of each island are different. Therefore, we show the individuals with different colors. The migration operation is performed periodically during the simulation process. There are many migration methods [42]. We show the flowchart of WMN-PSOSA-DGA in Figure 5.

### 3.4. Distribution of IoT Devices

**Figure 6:**
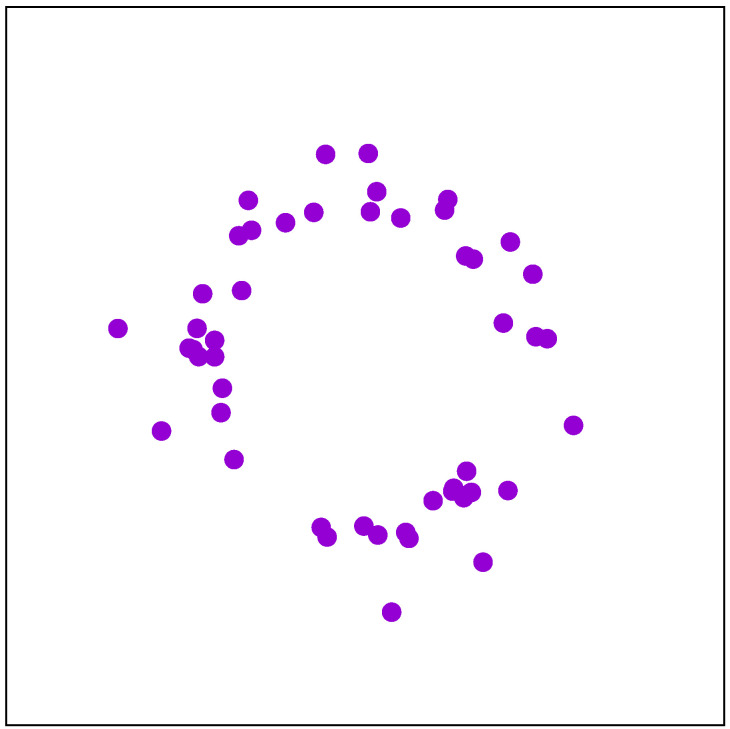
Stadium distribution.

WMN-PSOSA-DGA can generate many IoT device distributions. In this paper, we consider the IoT devices deployed as shown in Figure 6. We call this distribution Stadium distribution [43]. This distribution assumes that the usage of IoT devices happens in a stadium or around a pond.

## 4. Simulation Results

In this section, we show the simulation results. In this work, we analyze the performance of WMNs by using the WMN-PSOSA-DGA hybrid intelligent simulation system considering different replacement methods. We carried out the simulations 10 times in order to avoid the effect of randomness and create a general view of the results. We show the parameter setting for WMN-PSOSA-DGA in Table 2.

**Figure 7:**
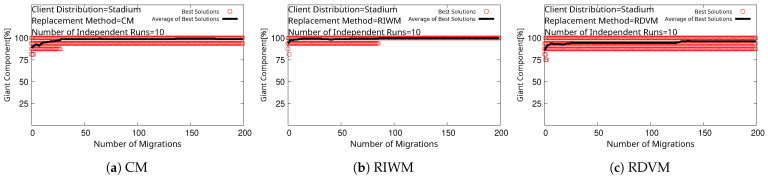
Simulation results of WMN-PSOSA-DGA for SGC.

**Figure 8:**
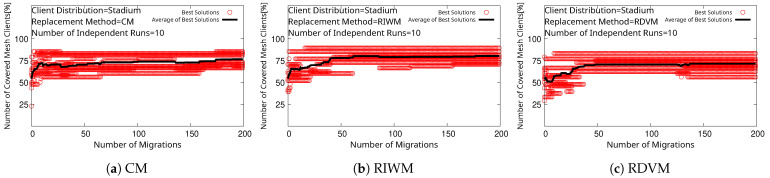
Simulation results of WMN-PSOSA-DGA for NCMC.

**Figure 9:**
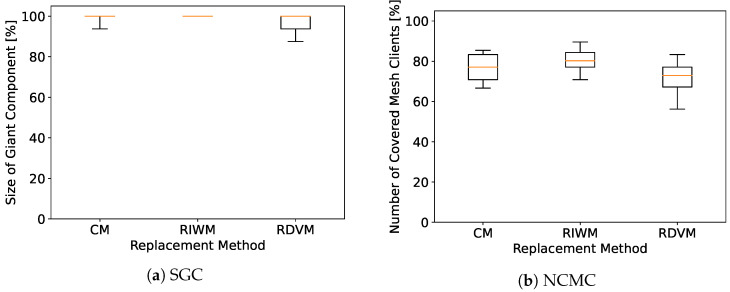
Simulation results of WMN-PSOSA-DGA.

We see that every replacement method converges to 100% for SGC, as shown in Figure 7. However, WMN-PSOSA-DGA does not reach 100% for NCMC in this case, as shown in Figure 8. We show boxplots of the results for each replacement method in Figure 9. We can see that RIWM has the highest performance compared to CM and RDVM.

## 5. Conclusions

In this work, we evaluated the performance of WMNs using a hybrid simulation system based on PSO, SA, and DGA (called WMN-PSOSA-DGA) considering the Stadium distribution of IoT devices. Since the performance of PSO depends on the method used to tune its parameters, we implemented three methods: CM, RIWM, and RDVM. The methods are compared for the results they achieve in optimizing the network toward two objectives: network connectivity and client coverage. Simulation results show that RIWM achieves the best performance compared to CM and RDVM.

While the simulations showed that the implemented methods achieve great effectiveness for the considered scenarios, they do not assure that the performance will remain high if deployed in a real environment. That is due to the fact that the system considers an ideal communication range of mesh routers and does not take into consideration the interference and the effect of the obstacles such as walls or other objects present in real environment. In future work, we will improve our hybrid intelligence in this direction and investigate the results the system can achieve for various combinations of the router replacement methods with selection, mutation, and crossover methods. 

## Figures and Tables

**Table 1:** Summary of research works with regards to the problem solved and the approach used.

Paper	Optimized Parameters	Approachd
[24]	Network connectivity, client coverage, and channel assignment	MILP and Relaxation-based Algorithm
[25]	Network connectivity and client coverage	GA
[26]	Network cost and client coverage	SA
[27]	Network connectivity and client coverage	PSO
[28]	Network connectivity and client coverage	PSO
[29]	Network connectivity and client coverage	PSO
[29]	Network connectivity and client coverage	PSO
This paper	Network connectivity and client coverage	PSO, SA, DGA

**Table 2:** WMN-PSOSA-DGA parameters.

Parameters	Values
Area size	32×32
Number of mesh routers	16
Number of IoT Devices	48
IoT device distribution	Stadium
Radius of a mesh router	2.0–3.5
Number of GA islands	16
Number of particle patterns	32
Number of migrations	200
Evolution steps	32
Selection method	Roulette wheel
Crossover method	SPX
Mutation method	Boundary
Crossover rate	0.8
Mutation rate	0.2
SA starting value	10.0
SA ending value	0.01
Total number of iterations	6400
Router replacement method	CM, RIWM, RDVM

## Data Availability

Not applicable.
